# Binding of Subdomains 1/2 of PfEMP1-DBL1α to Heparan Sulfate or Heparin Mediates *Plasmodium falciparum* Rosetting

**DOI:** 10.1371/journal.pone.0118898

**Published:** 2015-03-05

**Authors:** Davide Angeletti, Tatyana Sandalova, Mats Wahlgren, Adnane Achour

**Affiliations:** 1 Science for Life Laboratory, Department of Medicine Solna, Karolinska Institutet, Stockholm, Sweden; 2 Department of Microbiology, Tumor and Cell Biology (MTC), Karolinska Institutet, Stockholm, Sweden; University at Buffalo, UNITED STATES

## Abstract

The capacity of *Plasmodium falciparum* parasitized erythrocytes (pRBC) to adhere to the endothelial lining in the microvasculature and to red blood cells (RBC) is associated with the virulence of the parasite, the pathogenesis and development of severe malaria. Rosetting, the binding of uninfected RBC to pRBC, is frequently observed in individuals with severe malaria and is mediated by the N-terminal NTS-DBL1α domain of the adhesin *Plasmodium falciparum* erythrocyte membrane protein 1 (PfEMP1) expressed at the surface of the pRBC. Heparan sulfate has been suggested to be an important receptor for the NTS-DBL1α variant IT4_var60_ expressed by the parasite FCR3S1.2. Here, we have determined the binding site of NTS-DBL1α (IT4_var60_) to the RBC and heparin using a set of recombinant, mutated proteins expressed in and purified from *E*. *coli*. All the variants were studied for their ability to bind to RBC, their capacities to disrupt FCR3S1.2 rosettes, their affinities for heparin and their binding to rosette-disruptive mAbs. Our results suggest that NTS-DBL1α mediates binding to RBC through a limited number of basic amino acid residues localized on the surface of subdomains 1 (SD1) and 2 (SD2). The SD2-binding site is localized in close proximity to one of two previously identified binding sites in the rosetting PfEMP1 of the parasite PaloAlto-varO. The binding site in SD2 of NTS-DBL1α could represent a template for the development of anti-rosetting drugs.

## Introduction

*Plasmodium falciparum* malaria remains a major global health problem, with an estimated 600,000 deaths per year [1]. Rosetting, the binding of a parasitized red-blood cell (pRBC) to non-infected RBCs, is an important virulence factor that is associated with severe malaria [2–5]. It leads to micro-vascular obstruction in experimental models [6,7], a phenomenon that is also commonly associated with disease severity as seen in humans at autopsy [8–10].

The exported *Plasmodium falciparum* erythrocyte membrane protein-1 (PfEMP1) is hitherto the only identified parasite molecule that mediates rosetting [11]. Indeed, the sequence- and size-variation of the PfEMP1 molecules, which are encoded by approximately 60 *var* genes per genome [12], varies between 200–400 kDa and bestows the pRBC with a profound antigenic variation [13,14]. The different PfEMP1 variants share a common structural organization consisting of an N-terminal sequence (NTS) combined with tandem-arranged Duffy Binding Like domains (DBL) and Cysteine rich Inter-Domain Regions (CIDR) [11,12,15], positioned in a semi conserved domain cassette organization [15].

**Fig 1:**
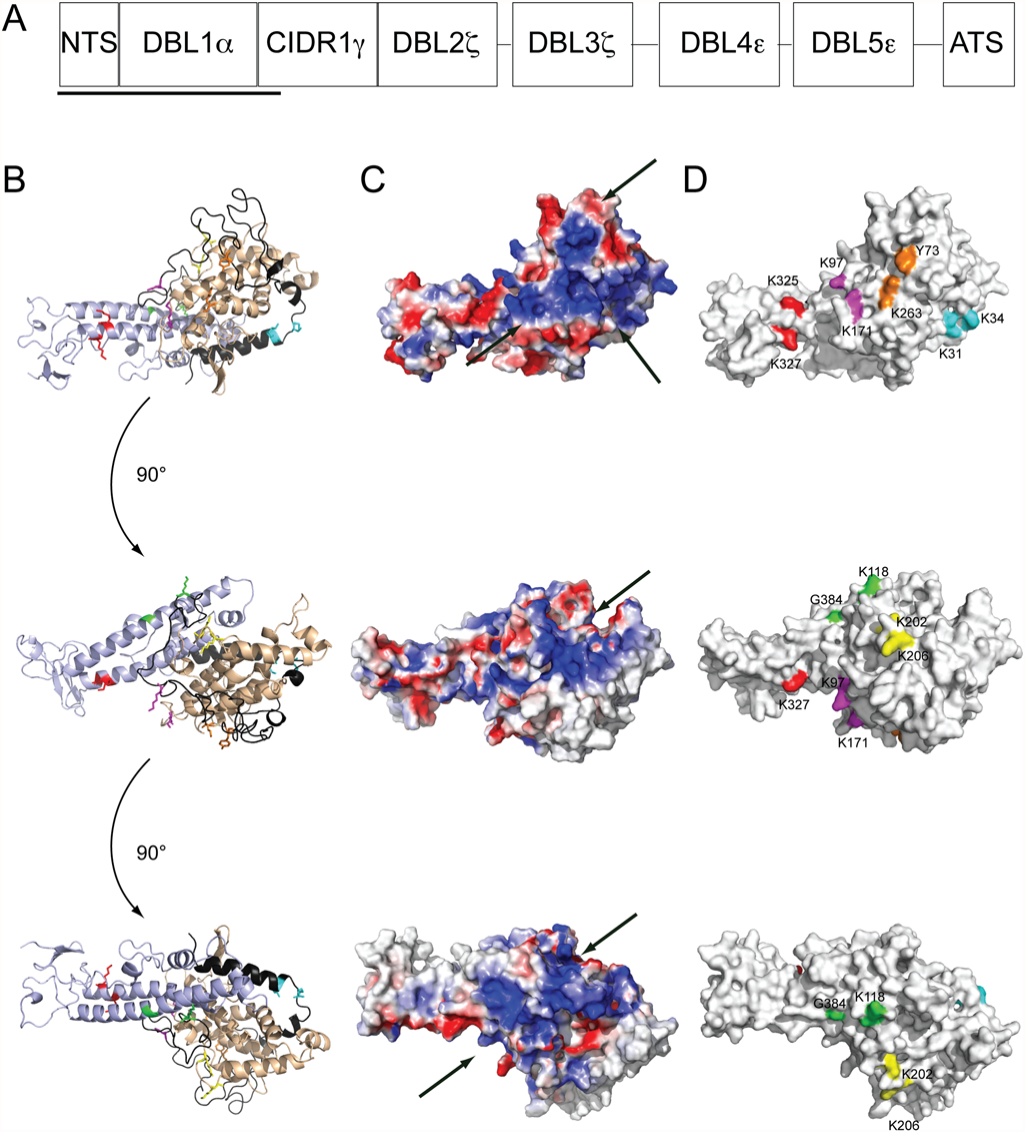
Localization of selected mutations on the protein molecular model. A) Domain organization of the IT4var60 PfEMP1 protein with underlined the construct used in this study. B) A molecular model of the NTS-DBL1α of IT4var60 was built based on the crystal structure of the NTS-DBL1α domain of PAvarO strain (PDB: 2yk0) [21]. Three 90 degrees orthogonal views of the molecule in the cartoon representation with mutated amino acids depicted in color. Subdomains 1, 2 and 3 are colored in black, light brown and light blue, respectively. C) Three 90 degrees orthogonal views of the molecule surface in the surface charge potential representation. Arrows indicate positively charged patches Blue: positive charge; white neutral charge; red: negative charge. D) Three 90 degrees orthogonal views of the molecule in the surface representation with mutated amino acids depicted in color. Mut A (Y73A, K263E): orange; Mut B (K118E, G384H): green; Mut C (K202A, K206A): yellow; Mut D (K97A, K171A): purple; Mut E (K325A, K327A): red; Mut F (K97A): black; Mut G (K263E): blue; Mut H (K31A, K34A): cyan.

The relatively conserved head structure of PfEMP1, and in particular the NTS-DBL1α domain, is crucial for mediating rosetting [16–18] (Fig. 1A). So far, heparan sulphate [19,20], blood group A and B tri-saccharides [21,22] and complement receptor-1 (CR1/CD35) [18,23] have been identified as rosetting-receptors on the RBC-surface. While the binding regions have been identified in these receptors [23,24], studies of the binding sites in NTS-DBL1α have until now focused only on the Palo Alto varO-NTS-DBL1α variant [21,25] while little is known about other rosetting PfEMP1s such as the IT4_var60_-variant of the rosetting parasite FCR3S1.2. Two binding-sites in the NTS-DBL1α of Palo Alto varO have been identified, one in subdomain 1 (SD1) which is suggested to mediate binding to heparin [25] and one on the opposite side of the NTS-DBL1α, that is suggested to mediate binding to the A-RBCs and blood group A tri-saccharide [21]. Indeed, an increased understanding of the interactions between the pRBC and the RBC during rosetting is of importance for the identification and design of compounds that could diminish the virulence of the parasite.

The aims of this study were to make use of the IT4_var60_ rosetting variant of PfEMP1 in order to establish if binding to receptors occurs through the use of a common, structurally conserved binding site. Furthermore, we have recently demonstrated that a large number of monoclonal antibodies (mAbs) generated against several rosetting laboratory strains target a common epitope, localized within the third subdomain (SD3) of NTS-DBL1α [26]. However, it remains unclear whether the efficiency of the neutralizing antibodies was due to direct blocking of the binding site or occurred through the induction of conformational changes in the targeted protein upon binding of the mAb.

Here the binding properties of the IT4_var60_-encoded NTS-DBL1α domain derived from the rosetting parasite FCR3S1.2 [27] were assessed, revealing that the RBC binding site is localized within the first and second subdomain (SD1 and SD2) of this molecule, in close proximity to the region previously identified in PAvarO as the RBC and blood group A binding site but at a distance from the PAvarO heparin-binding site [21,25]. In conclusion, the presented results suggest the possibility for a common RBC binding pattern shared between distinct rosetting variants. The findings presented here could be important for the future design of molecules that block rosetting.

## Results

### Design, expression, purification and biochemical characterization of mutated NTS-DBL1α (IT4var60) variants

A molecular model of NTS-DBL1α (IT4var60), including residues 1 to 483, was created based on the crystal structure of NTS-DBL1α (PAvarO) [21] (Fig. 1B). Since heparan-sulfate, the ligand targeted by the parasite on the surface of RBCs [24] is highly negatively-charged similarly to heparin, patches of positively charged residues were identified on the surface of NTS-DBL1α (IT4var60) (Fig. 1C). The patches were localized along opposite sides of the molecule spanning from the second (SD2) to the third subdomain (SD3) (Fig. 1B-D). Three patches were co-localized on one side of SD3 and the core of SD2, while the other two patches were localized on the opposite face of SD2. Key residues in each patch were selected for mutagenesis, based on their proximity, and altered to either alanine and/or residues with clearly opposite properties (Fig. 1 and Table 1). Two of the mutated proteins (Mut B and C) were localized within the positive regions that were limited to SD2. Conversely Mut A, D, E, F and G were localized in positive patches that span both SD2 and SD3. Finally, Mut H localized to the NTS region of the molecule where the heparin binding site for the PAvarO NTS-DBL1α was mapped [25] (Fig. 1B-C).

**Table 1:** Binding capacity and affinity of recombinant NTS-DBL1α (It4var60) variants to RBCs and ligands.

Name	Color code	Mutations	RBC binding	Rosette inhibition (IC_50_)(μM)	K_D_ to mAbV2–17.1 (nM)	K_D_ to heparin (μM)
wt	Black		++++	1.05	8.3±2.0	0.7±0.1
Mut A	Orange	Y73A K263E	No binding	No inhibition	9.7±1.4	0.8±0.1
Mut B	Green	K118E G384H	++	1.69	48.0±4.5	0.9±0.1
Mut C	Yellow	K202A K206A	++++	1.52	25.1±5.9	0.8±0.1
Mut D	Purple	K97A K171A	++++	0.96	14.4±2.7	2.2±0.2
Mut E	Red	K325A K327A	++++	1.02	4.8±0.7	0.9±0.1
Mut F	Grey	K97A	+	1.86	10.6±2.3	No Binding
Mut G	Blue	K263E	++	1.47	18.5±3.0	0.6±0.1
Mut H	Cyan	K31A K34A	++++	1.46	8.8±1.7	0.2±0.01

**Fig 2:**
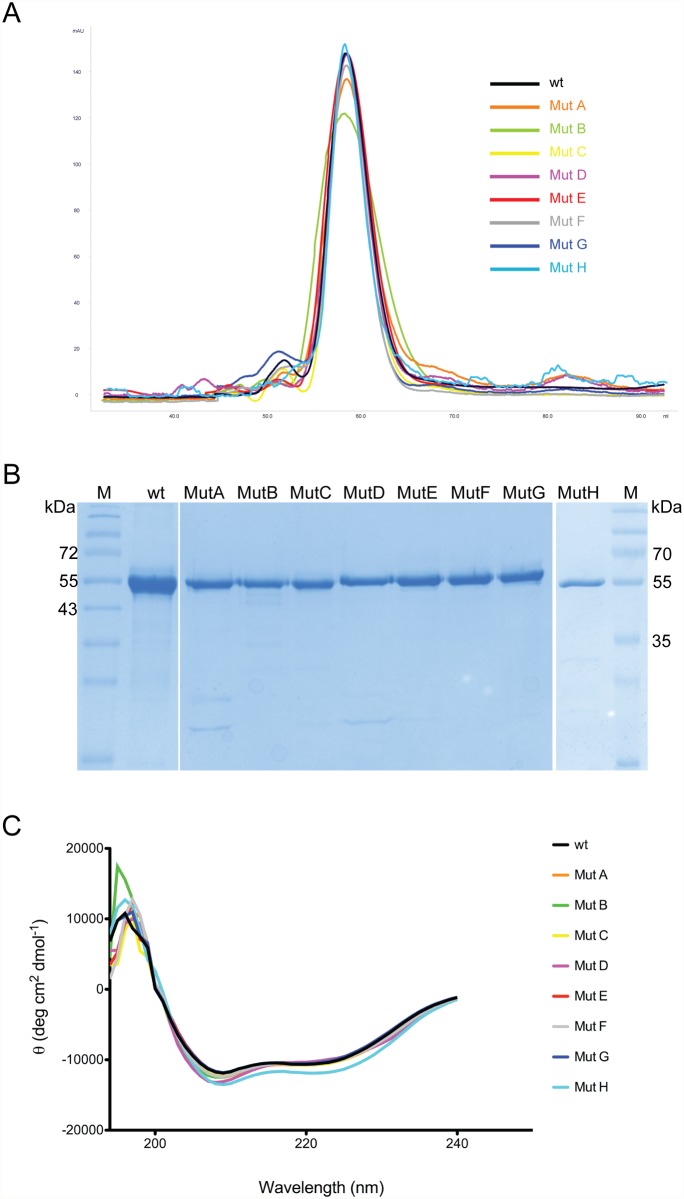
Expression of recombinant proteins in *E*. *coli*. A) Size exclusion chromatogram showing monomeric nature of wt and mutated proteins. B) The purity and quality of the mutants was assessed by electrophoresis: 2 μg of wt and mutant proteins were run on 12% SDS-PAGE gel under reducing conditions and stained with Coomassie. C) Far CD spectra of the proteins studied herein, showing nearly identical secondary structures for all the mutants. For color coding and mutation see Table I.

Wild type (WT) and all mutated forms of NTS-DBL1α (IT4var60) were produced as soluble proteins using an inducible bacterial expression system to yields higher than 2 mg/l. The size exclusion profiles indicated that all the produced proteins were monomers (Fig. 2A) and all purified proteins migrated as a single band in agreement with the predicted sizes (Fig. 2B). To assess the relative impact of the introduced mutations, all variants and WT-NTS-DBL1α (IT4var60) were evaluated for similar folding using circular dichroism (CD). Far UV CD spectra obtained for mutated NTS-DBL1α (IT4var60) proteins were similar to WT, indicating a close secondary structural identity (Fig. 2C). We interpret these data as evidence that all NTS-DBL1α (IT4var60) mutants used in the present study are structurally similar to WT-NTS-DBL1α (IT4var60). Furthermore, secondary structure predictions yielded no noticeable differences confirming the predominant α-helical structure of this protein, typical for the DBL fold (data not shown).

### Residues Y73, K97 and K263 of NTS-DBL1α (IT4var60) are essential for binding to RBCs

**Fig 3:**
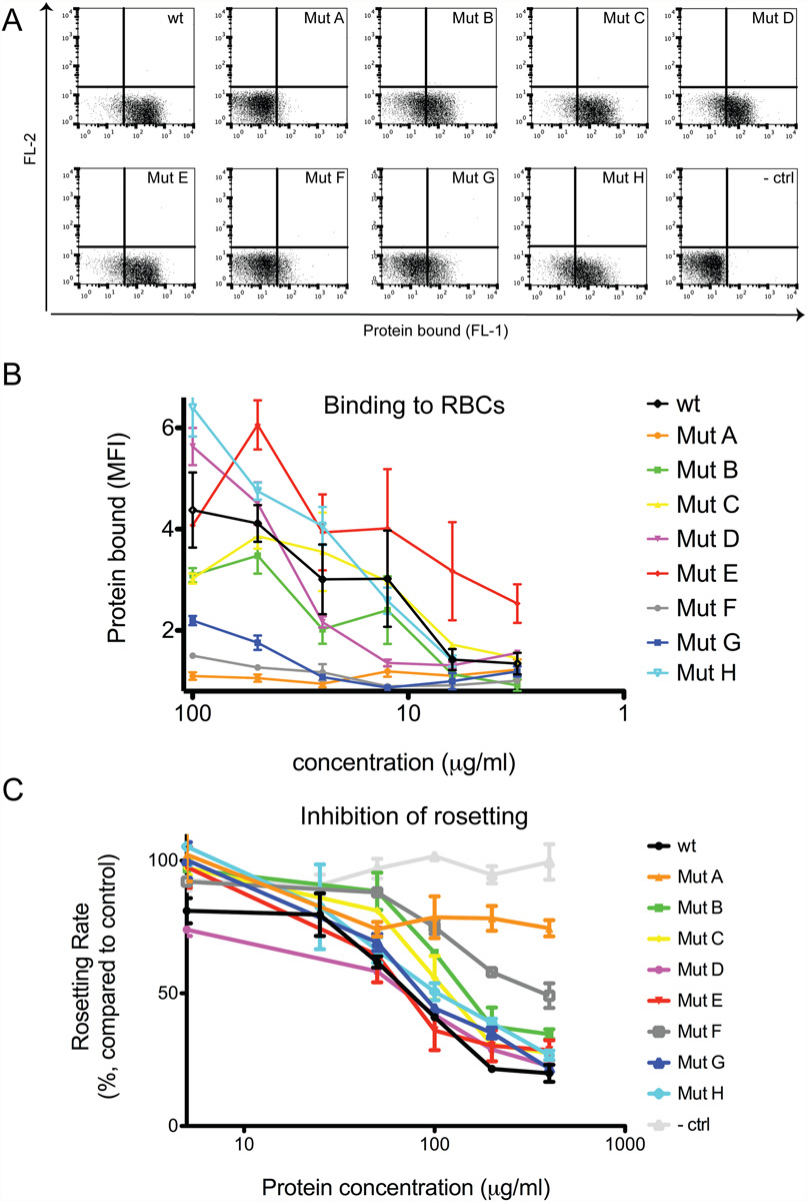
Functional activity of the mutated proteins. Mutant proteins were tested for their ability to bind O^+^ RBCs and disrupt FCR3S1.2 rosettes. A) Representative dot plot of protein binding to RBC at the highest concentration (100μg/ml) from flow cytometric analysis. B) Proteins were tested in serial dilution from 100μg/ml to 3.06μg/ml for binding to RBCs. Results are presented as mean fluorescence intensity (MFI) fold increase over a negative control. Three independent duplicate experiments were performed. C) FCR3S1.2 trophozoite rosettes were mechanically disrupted and allowed to reform in presence of different concentration of mutant proteins. Proteins were tested at different concentration ranging from 400μg/ml to 5μg/ml. Three independent experiments were performed in duplicate, results shown are average ± SEM.

The binding capacity of WT and NTS-DBL1α (IT4var60) variants to O^+^ RBCs was assessed using a flow cytometry-based assay, revealing that while WT-NTS-DBL1α (IT4var60) bound efficiently to RBCs in a dose-dependent manner, the binding of several variants was clearly impaired (Fig. 3A-B). In particular, substitution of residues Y73 and K263 to alanine and glutamate, respectively, abolished binding of Mut A to RBCs, even at high protein concentration. Interestingly, mutation of only residue K263 to a glutamate (Mut G) partially reduced binding to RBCs, suggesting that both Y73 and K263 are required for the formation of an adequate binding site (Fig. 3B).

Surprisingly, while mutation of residue K97 to an alanine in Mut F reduced binding by almost 80%, Mut D in which both K97 and K171 were substituted to alanine bound to RBCs with similar efficiency compared to WT-NTS-DBL1α (IT4var60) (Fig. 3B). Mutation of residues K118 and G384 to glutamate and histidine, respectively, impaired significantly the binding of Mut B to RBCs. In contrast, mutation of lysine 325 and 327 to alanine in Mut E increased binding affinity to RBCs. No effects were noticed when mutating two lysines (31 and 34), localized in the NTS. In conclusion, residues Y73, K97 and K263 play a key role in the interaction of WT-NTS-DBL1α (IT4var60) with RBCs.

### Mut A (Y73A, K263E) does not inhibit rosette reformation in the parasite FCR3S1.2

All recombinant proteins were tested for their ability to inhibit rosette reformation of the homologous parasite FCR3S1.2. While WT-NTS-DBL1α (IT4var60) efficiently inhibited rosette reformation in a dose-dependent manner, control NTS-DBL1α (TM284S2) that does not mediate rosetting, did not have any effect on the formation of rosettes (Fig. 3C). The rosetting frequency of the parasite, estimated to 85%, dropped upon incubation with the highest concentration of WT-NTS-DBL1α (IT4var60) to 14%. Overall, the ability of each mutated variant to inhibit rosetting corresponded well to their capacity to bind to RBCs, except for Mut G which surprisingly could inhibit rosetting despite poor RBC binding. In particular, the variant Mut A (Y73A, K263E) was unable to block rosetting (Fig. 3C). In conclusion, the rosette inhibition results confirmed the RBC binding assays, suggesting that binding of recombinant NTS-DBL1α engages the rosetting receptor on RBC surface.

### Mutation of residue K97 abolishes the capacity of NTS-DBL1α to bind to heparin

**Fig 4:**
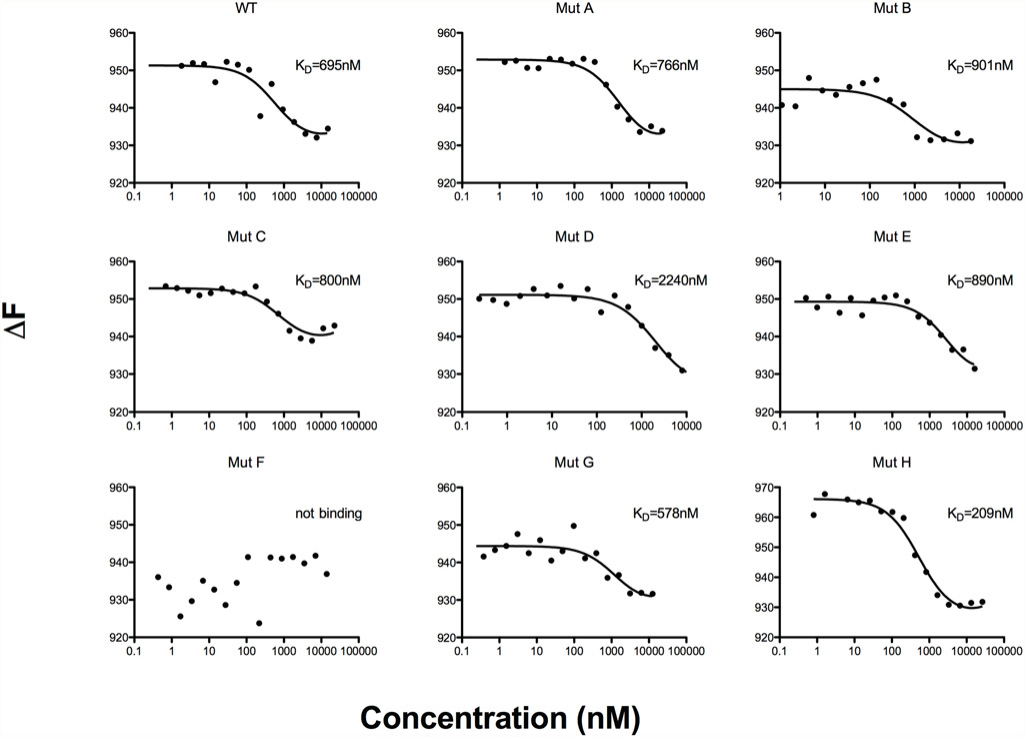
Mutated proteins binding and affinity to Heparin-FITC. Affinity of the mutated recombinant proteins for the ligand heparin was tested by microscale thermophoresis. Heparin FITC was kept at constant concentration of 100 nM while proteins were tested at ranging concentration varying between 0.5 to 60000 nM. Measurements were performed at 50% LED power and MST 60. Results were plotted using GraphPad Prism and K_D_ calculated using NanoTemper analysis software (Table 1).

While FCR3S1.2 is a blood group A-preferring parasite [22,28], heparan sulfate is the hitherto only known receptor on O^+^ RBCs [20,24]. Consequently, microscale thermophoresis was used to determine the affinity of each NTS-DBL1α variant to heparin. Concentration of FITC-conjugated heparin was kept constant at 100nM and the binding affinity was measured using a serial dilution of each protein. WT-NTS-DBL1α (IT4var60) bound to heparin with a K_D_ value of 700 nM, well in line with the previously reported affinity of the NTS-DBL1α domain from PAvarO (Table I) [25]. Surprisingly, Mut A (Y73, K263), which does not bind to RBCs, displayed similar affinity to heparin compared to WT- NTS-DBL1α (Fig. 4). Furthermore, most variants displayed unaltered affinity for the sugar. However Mut F (K97A) did not bind heparin and accordingly displayed markedly decreased RBC binding (Figs. 3B and 4).

**Fig 5:**
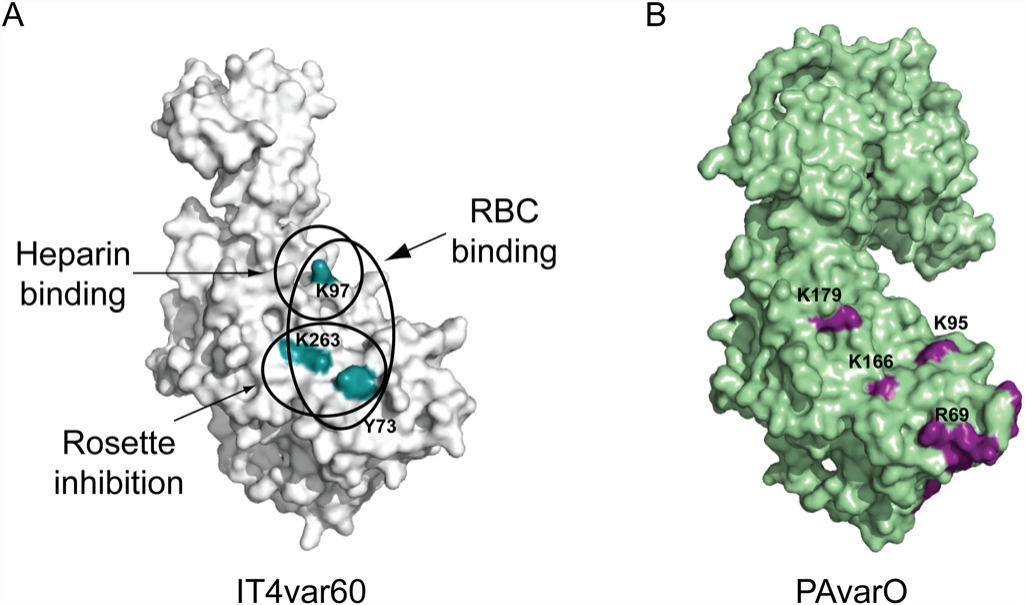
RBC and receptor binding site of NTS-DBL1α IT4var60. A) Molecular model of NTS-DBL1α IT4var60 showing the residues involved in RBC binding (Y73, K97 and K263), rosette inhibition (Y73 and K263) and heparin binding (K97). B) Comparison with previously identified residues that are important for BgA binding to the PAvarO variant (K179, K95, K166, R69: purple) [21].

In conclusion, four mutants affected RBC binding (Mut A, Mut B, Mut F, and Mut G), while two mutants reduced significantly heparin binding (Mut D and Mut F). Thus the presented heparin binding results do not exactly mirror the RBC binding results. Indeed, while Mut F (K97A) does not bind to heparin nor RBC, MutA (Y73A, K263E) displays reduced binding capacity to RBC but binds similarly to heparin compared to WT. The Mut A mutant completely abolished RBC binding and rosette disruption, but had no effect on heparin binding affinity. Conversely, Mut D displayed reduced heparin binding affinity by 3 folds, but had no effect on RBC binding activity or rosette disruption. Thus only one of the two established key mutations (Mut F with the K97A substitution) directly mirror the RBC binding results. Overall our results support the notion that the NTS-DBL1α domain may also bind to something else in addition to heparan sulfate on RBCs (Fig. 5).

### Rosette disrupting mAbs do not block the heparan sulfate/receptor binding site of NTS-DBL1α

We thereafter hypothesized that the reactivity of some NTS-DBL1α (IT4var60)-specific rosette-disruptive monoclonal antibodies (mAbs) could be directed towards the same region. We took advantage of two mAbs that have been extensively characterized [26], V2–3 which targets the α-helix 6 proximal to the SD3-loop of WT-NTS-DBL1α (IT4var60) and V2–17.1 whose conformational epitope remains undefined. Both antibodies disrupted efficiently parasite rosettes and blocked binding of WT-NTS-DBL1α (IT4var60) to RBCs [26]. It should also be noted that pre-incubation with the SD3-loop-specific antibody V2–3 partially inhibited binding of V2–17.1 to WT-NTS-DBL1α (IT4var60) [26], indicating possible overlapping epitopes. Both mAbs were tested against the panel of recombinant NTS-DBL1α (IT4var60) variants by ELISA, confirming that the V2–3 epitope is localized on the SD3-loop since mutation of lysine residues K325 and K327 in Mut E abolished binding to V2–3 (S1A Fig.). However, all the mutated variants retained similar binding capacity to V2–17.1 compared to WT-NTS-DBL1α (IT4var60) and the epitope for V2–17.1 remains undefined (S1A Fig.).

We hypothesized that despite not abolishing mAb binding, some of the introduced mutations could possibly reduce the affinity of the tested mAbs to NTS-DBL1α. While we were not able to assess the affinity of mAbV2–3 to NTS-DBL1α using microscale thermophoresis (data not shown), the affinity of V2–17.1 was in the low nanomolar range for WT NTS-DBL1α, with a K_D_ value of 8.3 nM (S1B Fig.). This is well in line with the affinity of most antibodies to their antigens. It should also be noted that Mut A (Y73, K263), which does not bind to RBCs, displays a similar level of affinity for V2–17.1 (K_D_ 9.7 nM), confirming the ELISA results, and demonstrating that V2–17.1 does not bind directly to the receptor binding site of NTS-DBL1α (IT4var60). All the other tested variants maintained a high affinity interaction with V2–17.1, all within the nM range (Table 1 and S1B Fig.). In summary our results demonstrate that the binding of mAbV2–17.1 to NTS-DBL1α variants is of high affinity and is not affected by any of the mutations hereby studied.

## Discussion

Although the association between the capacity of *P*. *falciparum* to form rosettes and severe disease is now well-established [2,4], still remarkably little is known about the exact composition of NTS-DBL1α residues that are involved in these interactions, essential for parasite virulence. The generation of strain-transcending antibodies, which would target multiple rosetting parasite variants, has hitherto proven to be challenging [29,30]. Conversely, recent successes suggest that it is possible to raise cross-reactive antibodies, although only to restricted subgroups of parasites with conserved binding signatures [31,32]. Given the large number of rosetting receptors [33], the identification of a shared ligand-binding site would significantly improve the possibility to design drugs that would inhibit a larger panel of parasite strains and/or improve other non-excluding strategies that focus on Ab responses towards conserved regions of PfEMP1. In the present study, we have identified the RBC binding site of the NTS-DBL1α domain that is expressed on the surface of the FCR3S1.2/IT4var60 parasite line. We also demonstrate that mAbs that are able to disrupt rosettes do not block directly the receptor binding site of WT-NTS-DBL1α (IT4var60).

The NTS-DBL1α domain of PfEMP1 plays a key role in rosetting [16–18,24]. In order to establish the fine molecular details underlying binding we generated a set of mutations localized in positively charged patches. All the purified variants were monomeric with equivalent fold to WT-NTS-DBL1α (IT4var60). Substitutions of residues Y73 and K263 in Mut A abolished binding to RBCs and the capacity to disrupt FCR3S1.2 rosettes, demonstrating the crucial role of this positively charged patch for binding to RBCs. Further residue K97 was also identified as a key mediator for binding to heparan sulfate/heparin and RBCs. These three residues are localized proximally on the surface of the molecule forming a binding site for the red cell receptor heparan sulfate/heparin and possibly composing a high affinity receptor binding site that is important for rosetting (Fig. 5). Since residues Y73 and K263 do not affect heparin binding it is tempting to speculate that the parasite could make use of multiple receptors for RBC binding. Furthermore it should be noted that residues Y73 and K263 are also in close proximity to the region formed by residues R69, K95, K166 and K179, which has been proven to be crucial for RBC binding by NTS-DBL1α of PAvarO (Fig. 5B) through interaction with the BgA receptor [21]. Strikingly, despite the fact that the two parasites make use of different ligands [20,21], the respective binding sites appear to be localized in very close proximity, suggesting a possible conserved binding mechanism among distinct rosetting parasite strains. Vigan-Womas *et al*. have also demonstrated that the main binding site for the BgA trisaccharide also comprises several other residues, all localized proximally to the tetrameric patch formed by residues R69, K95, K166 and K179. In contrast, the binding site of IT4var60 seems to be more restricted to a few residues. Indeed, binding of Mut C (K202 and K206), which comprises equivalent residues to those demonstrated as critical for PAvarO for RBC binding, is not affected. It should be noted that a proximal region in subdomain 2 is also targeted by strain-transcending antibodies [31].

The heparin-binding site of IT4var60 is localized in SD2 while the PAvarO NTS-DBL1α is site on the opposite face of the molecule, in the NTS (S2 Fig.). Study of individual DBL-domains interaction with charged substrate can be difficult due to the presence of promiscuous and non-specific binding [34,35], which could explain some of the discrepancies between RBC binding/rosette disruption and heparin binding data. However other studies have previously demonstrated specificity in DBL domains binding to heparin [25,36]. In our study, mutations to lysines 31 and 34 (Mut H), localized in the NTS, did not affect the ability of the recombinant protein to bind neither heparin nor RBCs. This result is not surprising since heparan sulfate is a receptor for FCR3S1.2 [20,24] but not for PAvarO parasite [25]. Binding of Mut F (K97A) to RBCs was significantly reduced compared to WT, corroborating results from previous studies and confirming the role of heparan sulfate as a rosetting receptor for this parasite strain [20,24]. However, since Mut A (Y73A/K263E) maintained unaltered affinity for heparin compared to WT, it is possible that NTS-DBL1α makes use of multiple receptors for rosetting. Presence of several receptors is supported by previous studies on FCR3S1.2 parasite [16,28] as well as on PAvarO, that utilizes both blood group A sugar and an unidentified receptor on O RBCs as rosetting ligand [21].

In this study we have directed our attention to the O^+^ RBC receptor heparin/heparan sulfate; however we have not tested binding of the recombinant protein to the other known rosetting receptor complement receptor 1 [18]. In addition, while it is known that FCR3S1.2 is a BgA preferring parasite [22,28], recent data from our laboratory indicate that NTS-DBL1α is not the main BgA ligand (Goel et al, manuscript in preparation).

We confirmed the epitope of mAbV2–3, which has been previously mapped using peptide arrays [26]. However we were not able to identify the conformational binding site of mAbV2–17.1. We may speculate for the existence of a common mechanism of action for rosette-inhibiting antibodies through conformational changes within distance from the paratope. Such an effect has previously been reported for DBP, where adhesion-blocking mAbs target a region within SD3 [37] while the receptor-binding site is localized at the dimer interface, in a region similar to the one reported herein for NTS-DBL1α of IT4var60 [38,39]. Focusing the immune response to a distal variable site, as compared to the receptor-site, could represent a mechanism that parasites use in order to divert the immune system and hamper the production of strain-transcending antibodies, a mechanism shared by several other pathogens [40]. Co-crystallization of a Fab fragment from such a mAb and of the targeted domain would verify this hypothesis. Unfortunately, despite numerous efforts, we have hitherto not been able to achieve this goal.

In conclusion we report here the identification of the RBC binding site of the rosetting domain NTS-DBL1α of IT4var60. We demonstrate that it is localized in proximity to the blood group A binding site of NTS-DBL1α of PAvarO suggesting a possible structurally conserved binding site between rosetting strains and opening new possibilities for therapeutic interventions against severe malaria.

## Materials and Methods

### Protein expression and purification

NTS-DBL1α encoded by IT4var60 was used to design a synthetic gene, starting from amino acid 1 to 483 and synthesized by DNA 2.0 (USA) into pJ414 expression vector with His-tag. The codon optimized sequence has been deposited in GenBank, accession number KP296175. Proteins were expressed and purified as described previously [29]. Briefly, protein was expressed in *E*. *coli* SHuffle T7 Express *lisY* (New England Biolabs); bacteria were grown at 30°C until OD_600_ = 0.6 and then induced with 0.4μM IPTG (Sigma Aldrich) for 20 hours at 16°C. Protein was extracted in Sorbitol buffer (20% Sorbitol, 150mM NaCl, 20mM Hepes pH7.4), subjected to osmotic shock (5mM MgSO_4_) and lysed by sonication in 20% Glycerol, 150mM NaCl, 20mM Tris pH8. Purification was carried out by metal affinity chromatography (TALON, Clontech) followed by size exclusion chromatography (Superdex 75 16/60, GeHealthcare). NTS-DBL1α from TM284S2, used as negative control, was expressed in *E*. *coli* BL21(DE3) and purified from inclusion bodies as described [29].

### Mutagenesis

Point mutations were introduced using PCR based mutagenesis kit (Quickchange Site-Directed mutagenesis kit, Stratagene) following the manufacturers instructions using the recoded gene of NTS-DBL1α in the plasmid pJ414 as template. All mutants were expressed and purified as described above.

### Circular dichroism

CD spectra were measured using Jasco J-810 spectrometer (Jasco) with the proteins in PBS pH7.4 at 0.3–0.8 mg/ml. Far UV spectra was recorded between 190 and 260 nm using a cell with 0.05cm path length and 0.2nm steps with averaging time of 4s per step. The scan was repeated for three consecutive times and averaged to obtain the spectrum. Obtained spectra were corrected against buffer spectrum measured in the same conditions and converted to mean residue ellipticity by normalizing for concentration, path length and mean reside weight. Secondary structures were estimated using the CDSSTR method from the Dichroweb server [41].

### Protein binding assay to RBCs

Protein binding assay was performed as described previously [26]. Recombinant proteins (from 100μg/ml to 3.06μg/ml, serially diluted 1:2) in PBS were incubated for 30´ with O^+^ RBCs at RT. Detection of bound protein was performed by flow cytometry (FACScan, Becton Dickinson) after fluorescent labeling with mouse α-his mAb (0.5μg/ml, QIAGEN) followed by α-mouse-alexa488 conjugated (1:100, Invitrogen). 100000 RBCs were counted per sample. In order to correct for variations in between experiments results are presented as a fold increase (ratio) of mean fluorescence intensity (MFI) over negative control. Flowjo (Tree Star) was used for data analysis.

### Parasite culture

Culture of parasites was carried out according to standard methods in 10% serum with gassing and orbital shaking [42]. Enrichment of pRBCs on mAb in order to maintain monovariant FCR3S1.2 culture was performed routinely [17].

### Inhibition of rosette reformation

Rosetting FCR3S1.2 trophozoites (24–30 hours p.i.; rosetting rate between 75 and 90%) with parasitemia between 2 and 8% were used for the experiments. Parasites were spun down, washed and resuspended in RPMI (Gibco). Rosettes were mechanically disrupted using a syringe with 0.6mm diameter blunt needle (Kendall) and complete disruption confirmed by microscopy. Recombinant protein in PBS was aliquoted in a range of concentration (from 800μg/ml to 2μg/ml) into a 96 well plate and serum was added at 20%; finally, disrupted parasite culture was added 1:1 to each well and incubated at room temperature for 30´. A drop from the well was mixed with acridine orange and applied on a microscopy slide. pRBC bound to two or more non-infected RBCs was count as a rosette. Counting was performed diagonally through the slide with minimum 25 fields counted per slide with two slides per well. Rosetting rate is presented as % relative to a control incubated with PBS.

### ELISA

Reactivity of two of the mAbs generated in previous study [26] were tested in ELISA against the mutant proteins as described [29]. Briefly, plates were coated overnight with 1μg/ml of protein, subsequently blocked and incubated with mAbs containing solution in serial ten fold dilution from 1 to 0.01 mg/ml. Secondary goat anti-mouse Ab ALP conjugated was used at 1:1000 dilution and reaction developed using SIGMA*FAST* p-Nitrophenyl phosphate tablets (Sigma).

### Microscale thermophoresis

Microscale thermophoresis experiments were performed using Monolith NT 115 (NanoTemper Technologies) and analyzed using NanoTemper Analysis software. The principles of the method are described elsewhere [43]. mAbs were labeled with the fluorescent dye NT-547 using the Monolith NT Protein labeling kit Green-NHS (amine reactive) according to manufacturer´s instructions (NanoTemper Technologies). The unlabeled NTS-DBL1α WT and variants were serially diluted from 2μM to 35pM and incubated for 30 minutes 1:1 with 10nM of labeled mAb. For heparin binding, the unlabeled NTS-DBL1α WT and variants were serially diluted from 60μM to 0.2nM and Heparin-FITC was used at 100nM. All experiments were performed in standard capillaries (NanoTemper Technologies) at least in duplicates. Each samples was run at three different MST powers: 40, 60 and 80. All experiments were run in PBS pH7.4 + 0.05% Tween-20.

## Supporting Information

S1 Fig Binding and affinity of mAbs to recombinant proteins. A) Two mAbs with different specificities were tested for binding of mutated proteins. 1μg/ml of protein was coated per well, assayed with different concentration of mAbs and detected with anti-mouse ALP conjugated. Presented are results at 10μg/ml of mAbs. Results are mean of three independent experiments in duplicate ± SEM. B) Affinity of mAbV2–17.1 for the mutated constructs was assayed by microscale thermophoresis. mAb was labeled with the fluorescent dye NT-547 and its concentration kept constant at 10nM. Recombinant proteins were tested at 14 different concentrations between 0.02 and 250nM. Measurements were performed at 70% LED power and MST 80. Results are mean of two independent thermophoresis measurements presented as mean ± SEM. Results were plotted using GraphPad Prism and K_D_ calculated using NanoTemper analysis software (Table 1).
(TIFF)

S2 Fig Comparison of the heparin binding sites of NTS-DBL1α PAvarO and SD1 of IT4var60. A) Comparison of the mutation in SD1 (K31 and K34, Mut H in cyan) of IT4var60 with the identified heparin binding site of PAvarO (K20, K32, K40, K423, K424, K451, K456). The mutated residues are not exactly in the corresponding position because the NTS-DBL1α of IT4var60 lacks lysine residues in the corresponding position. B) Surface charge potential representation shows that PAvarO has a positively charged patch corresponding to the heparin binding site, which is lacking in the IT4var60 molecule. Blue: positive charge; white neutral charge; red: negative charge.
(TIF)
