# Supplementary figures and images for: Binding of Subdomains 1/2 of PfEMP1-DBL1α to Heparan Sulfate or Heparin Mediates Plasmodium falciparum Rosetting
Source: PLoS One. 2015 Mar 5;10(3):e0118898. doi: 10.1371/journal.pone.0118898 (PMC4351205; doi:10.1371/journal.pone.0118898)

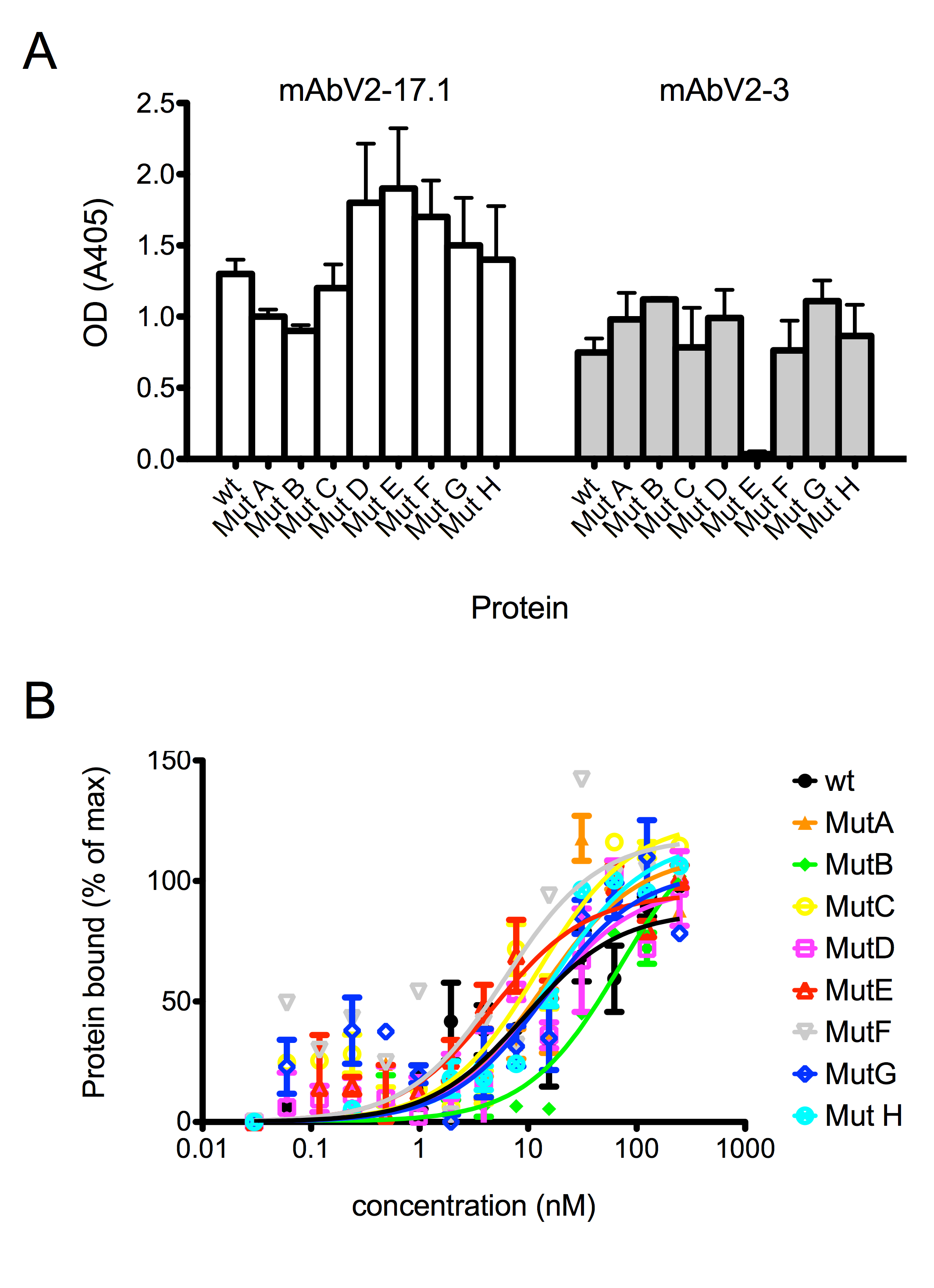

Supplement: S1 Fig — A) Two mAbs with different specificities were tested for binding of mutated proteins. 1μg/ml of protein was coated per well, assayed with different concentration of mAbs and detected with anti-mouse ALP conjugated. Presented are results at 10μg/ml of mAbs. Results are mean of three independent experiments in duplicate ± SEM. B) Affinity of mAbV2–17.1 for the mutated constructs was assayed by microscale thermophoresis. mAb was labeled with the fluorescent dye NT-547 and its concentration kept constant at 10nM. Recombinant proteins were tested at 14 different concentrations between 0.02 and 250nM. Measurements were performed at 70% LED power and MST 80. Results are mean of two independent thermophoresis measurements presented as mean ± SEM. Results were plotted using GraphPad Prism and KD calculated using NanoTemper analysis software (Table 1). (TIFF) [file pone.0118898.s001.tiff]

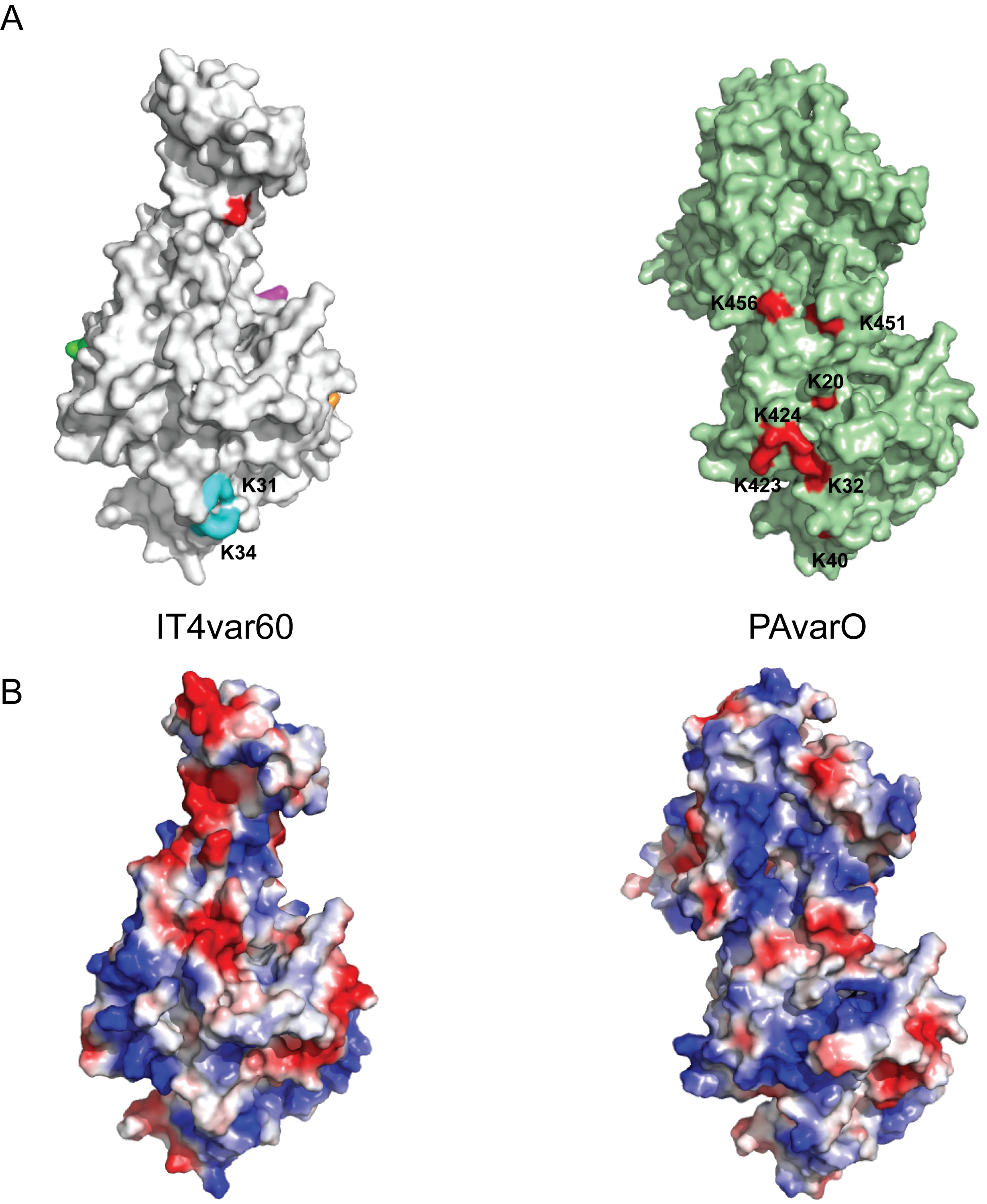

Supplement: S2 Fig — A) Comparison of the mutation in SD1 (K31 and K34, Mut H in cyan) of IT4var60 with the identified heparin binding site of PAvarO (K20, K32, K40, K423, K424, K451, K456). The mutated residues are not exactly in the corresponding position because the NTS-DBL1α of IT4var60 lacks lysine residues in the corresponding position. B) Surface charge potential representation shows that PAvarO has a positively charged patch corresponding to the heparin binding site, which is lacking in the IT4var60 molecule. Blue: positive charge; white neutral charge; red: negative charge. (TIF) [file pone.0118898.s002.tif]
